# Knee Chest Position as a Sign of Pericarditis

**Published:** 1911-08

**Authors:** 


### Knee Chest Position as a Sign of Pericarditis

M. Edgar Hertz, of the Necker Hospital, Paris, (*La Tribune Medicale,* May, 1911), cites four cases of pericarditis with marked effusion in which relief of the dyspnea was secured by the knee chest posture, by the spontaneous endeavors of the patient himself to find a comfortable position. He knows of no other condition in which the patient assumes this posture of his own accord. The action seems to be purely mechanic and not due to the exact pathologic lesion as the sign has been noted in rheumatic, tuberculous, post-scarlatinal and nephritic pericardial effusion.

We would add the caution that this sign is merely suggestive; also that when the knee chest posture is not spontaneously assumed by the patient it might be well to place him in it, if necessary with a pillow or strap support, both for diagnostic and therapeutic purposes.

